# The Preparation of the Compound Syrup of Phosphates or Chemical Food

**Published:** 1859-01

**Authors:** Phil. F. Mahla

**Affiliations:** Apothecary, Chicago


ARTICLE V.

### THE PREPARATION OF THE COMPOUND SYRUP OF PHOSPHATES OR CHEMICAL FOOD.

BY DR. PHIL. F. MAHLA, APOTHECARY, CHICAGO.

The favorable results which have been obtained in medical practice by the use of the so-called "chemical food," made it desirable to find out a method by which this elegant medicament could be made in a manner which would permit every apothecary to put it up himself, and which would, by its simplicity, reduce the cost of its preparation.

Different formulas have been proposed for preparing the soluble phosphates. The most common was that in which the basic phosphates were dissolved in pure glacial phosphoric acid, by which simply the soluble acid phosphates were formed, or that in which another acid was used, whereby, however, besides the soluble phosphates, also the soluble salt of the employed acid was formed in solution.

These procedures were not only expensive ones, because in most cases instead of the costly glacial phosphoric acid the nearly just as high priced citric acid has been used, but they did even not furnish the desired thing, because the physician wants only the pure phosphates without free phosphoric acid in solution. But to prepare the syrup after the heretofore published formulas, it is not possible to get it without having free acid in it.

I think, therefore, that it would be of service to the medical profession, and especially to my brethren in business, if I would make public my process, which permits not only to get up a pure and elegant preparation, but which brings down the cost to a mere trifle; thus affording the apothecary an opportunity to charge but low prices for a valuable medicine.

The calcined bones consist essentially of about 90 per cent tribasic phosphate ($3CaO.PO^5$) and about 10 per cent carbonate of lime. If they are brought in contact with sulphuric acid, a chemical process is produced, by which, besides the monobasic phosphate of lime $\left(\begin{smallmatrix}CaO.\\2HO\end{smallmatrix} + PO^5\right)$, only the but slightly soluble sulphate of lime is formed. If the obtained solution, after the insoluble residue has been separated, is evaporated to dryness, and the remaining salts are treated with a small quantity of water, nearly all the sulphate of lime remains as an insoluble white powder, and the solution contains the monobasic phosphate of lime.

I found now that this solution is capable of dissolving a large quantity of phosphate of iron, and that it can keep it in solution if it is not heated, provided that an excess of the monobasic phosphate of lime has been used. My method is founded on this discovery.

I will first give the formulas for preparing the monobasic phosphate of lime and the phosphate of iron, and then the one for making the syrup.

MONOBASIC PHOSPHATE OF LIME.—Take of powdered calcined bones ℥xvi., pour on them, in a porcelain evaporating dish of convenient size, one gallon of water, and add, stirring constantly, by and by, sulphuric acid (oil of vitriol) ℥x. After the effervescence has ceased, heat the mass on a charcoal fire, and keep it boiling during two or three hours, stirring constantly, and adding from time to time so much water as has been evaporated. Strain the refrigerated mass, and mix the residue twice with a quart of water and strain again. Evaporate to dryness all the filtering and wash-waters, first on free fire, and afterwards on the waterbath, dissolve the remaining mass in an equal quantity of cold water, and after having separated the insoluble sulphate of lime by filtering, evaporate the clear liquid to a very thick pill mass consistence or better dryness. Put in dry glass-stopper bottles and protect it from the air.

Phosphate of Iron.—Dissolve of sulphate of iron 10 parts in 100 parts of water, add to it a solution of 13 parts of phosphate of soda in 130 parts of water (both solutions have to be cold), pour away the supernatant liquor as soon as the precipitate has settled, wash it with cold water, and collect it finally on a filter. Then dissolve one ounce of the monobasic phosphate of lime (if of pill mass consistence, one scruple more), one drachm of the phosphate of soda, and one drachm of the phosphate of potash, in four ounces of distilled water; add to this solution the still moist and freshly made phosphate of iron (out of about 10 drachms of the sulphate) as long as it will dissolve. As soon, however, as the blue color of the latter ceases to disappear, add another ounce of monobasic phosphate of lime, and filter the solution into a bottle. All these operations have to be made cold without heating the liquid.

Take of white sugar 32 ounces, water 18 ounces; boil and strain. When this syrup is cooled add to it the above solution.

This syrup contains in 1 drachm, 2 grains of the monobasic phosphate of lime, 1 grain of the phosphate of iron, and smaller proportions of the phosphates of soda and potash.

It happens very often that a small percentage of lime, which is contained in nearly all sugars, causes a turbidness in the ready-made clear syrup by throwing down a precipitate of the basic phosphate of lime. By letting it deposit, or better by filtering through a good filtering paper, it can be obtained perfectly clear.

A few druggists color this syrup with cochineal, but this being of no other use than to give this preparation the disagreeable taste of this substance, I think it preferable not to do it.

